# The International Society of Surgery

**Published:** 1905-09-30

**Authors:** 


### The International Society of Surgery.

The disorder and discomfort attending the later meetings of the International Congress of Medicine might be thought to be arguments against the formation of any further International Congresses. But the conduct of the first Congress of the International Society of Surgery shows that such a gathering can be held with a maximum of profit and a minimum of discomfort. The International Society of Surgery was founded in Brussels in 1902, and the Congress held last week was practically the first meeting at which members could obtain an interchange of ideas upon the various subjects chosen for discussion. The meeting was patronised by H.M. the King of the Belgians and his Minister of Agriculture. It was under the presidency of Prof. Kocher, of Berne, and was attended by no less than 217 surgeons out of a total of 700 members. The Palais des Académies at Brussels formed an adequate meeting-place, and credit is due to the executive, with Dr. Depage as secretary, for the admirable manner in which all the arrangements were carried out. The subjects for discussion had been chosen with care, and a series of reports written by competent authorities was in the hands of the members some weeks before the actual meeting. Arrangements had been made at the hotels to suit the pockets of all comers, and the lighter side of a congress in the form of a banquet, a gala performance at the theatre, and a reception in the magnificent Hôtel de Ville, was not neglected. It is not surprising, therefore, that these attractions led many members to bring their wives with them, and, as there was much friendly intercourse between the ladies of the different countries, the social uses of the Society were well brought out.

The Society has wisely determined to limit the number of its members, so that it might not become unwieldy. It is to meet once every three years at Brussels, because that town is central and easy of access to all European nations. The official languages of the Congress are to be French, German, and English; and, from the experience gained at the meetings held last week, it was evident that three languages will be sufficient.

The government is to be in the hands of a president—on the next occasion Professor Czerny, of Heidelberg—and an International Committee. The Committee is formed by delegates from each nation; and from the members of each nationality three are chosen to form a National Committee. The delegate is to be selected from these three members. The triennial subscription of forty-five francs has been reduced to forty francs in consequence of the large increase of members. The work of English secretary has hitherto been most ably performed by Mr. Reginald Harrison. The Congress re-elected him as a representative of surgery in Great Britain, and associated with him Mr. Edmund Owen and Mr. D'Arcy Power. It is difficult to organise a meeting where there are many members, most of whom are anxious to take part in the discussion, and although the Brussels meetings were distinctly well organised, there were many small details which the experience of the last week will show to be capable of improvement at the next meeting in 1908. The method of procedure followed at our large medical gatherings might well be followed somewhat more closely, for experience has taught us many things, and not least what it is best to avoid.

